# Beneficial effects of running exercise on hippocampal microglia and neuroinflammation in chronic unpredictable stress-induced depression model rats

**DOI:** 10.1038/s41398-021-01571-9

**Published:** 2021-09-06

**Authors:** Kai Xiao, Yanmin Luo, Xin Liang, Jing Tang, Jin Wang, Qian Xiao, Yingqiang Qi, Yue Li, Peilin Zhu, Hao Yang, Yuhan Xie, Hong Wu, Yong Tang

**Affiliations:** 1 Department of Histology and Embryology, Chongqing Medical University, 400016 Chongqing, P. R. China; 2 Laboratory of Stem Cells and Tissue Engineering, Chongqing Medical University, 400016 Chongqing, P. R. China; 3 Department of Physiology, Chongqing Medical University, 400016 Chongqing, P. R. China; 4 Department of Pathophysiology, Chongqing Medical University, 400016 Chongqing, P. R. China; 5 Department of Radioactive Medicine, Chongqing Medical University, 400016 Chongqing, P. R. China; 6 Institute of Life Science, Chongqing Medical University, 400016 Chongqing, P. R. China; 7 Department of Biomedical Engineering, Chongqing Medical University, 400016 Chongqing, P. R. China

**Keywords:** Depression, Hippocampus

## Abstract

Running exercise has been shown to relieve symptoms of depression, but the mechanisms underlying the antidepressant effects are unclear. Microglia and concomitant dysregulated neuroinflammation play a pivotal role in the pathogenesis of depression. However, the effects of running exercise on hippocampal neuroinflammation and the number and activation of microglia in depression have not been studied. In this study, rats were subjected to chronic unpredictable stress (CUS) for 5 weeks followed by treadmill running for 6 weeks. The depressive-like symptoms of the rats were assessed with a sucrose preference test (SPT). Immunohistochemistry and stereology were performed to quantify the total number of ionized calcium-binding adapter molecule 1 (Iba1)^+^ microglia, and immunofluorescence was used to quantify the density of Iba1^+^/cluster of differentiation 68 (CD68)^+^ in subregions of the hippocampus. The levels of proinflammatory cytokines in the hippocampus were measured by qRT-PCR and ELISA. The results showed that running exercise reversed the decreased sucrose preference of rats with CUS-induced depression. In addition, CUS increased the number of hippocampal microglia and microglial activation in rats, but running exercise attenuated the CUS-induced increases in the number of microglia in the hippocampus and microglial activation in the dentate gyrus (DG) of the hippocampus. Furthermore, CUS significantly increased the hippocampal levels of inflammatory factors, and the increases in inflammatory factors in the hippocampus were suppressed by running exercise. These results suggest that the antidepressant effects of exercise may be mediated by reducing the number of microglia and inhibiting microglial activation and neuroinflammation in the hippocampus.

## Introduction

Major depressive disorder (MDD) is a common mental disorder characterized primarily by symptoms of pervasive depressed mood along with anhedonia and feelings of guilt [1]. It is estimated that the aggregate prevalence and lifetime risk of depression are approximately 12.9 and 10.8%, respectively [2]. As the leading cause of disability and suicide among psychiatric disorders, MDD severely affects patients’ quality of life and increases the global disease burden [3, 4].

Although the exact etiology and pathogenesis of depression remain unclear, increasing evidence has shown that the inflammatory response is a main factor involved in the development of the disorder [5, 6]. Numerous clinical studies have verified that the circulating levels of proinflammatory cytokines in depressed individuals are higher than those in nondepressed subjects [7, 8]. In addition, chronic mild stress substantially increases the risk of elevated inflammatory cytokine levels in the brains of rats [9]. The hippocampus is the key structure responsible for the regulation and control of emotion [10]. Postmortem studies have provided evidence that several inflammatory genes are upregulated in the hippocampi of MDD patients [11], and the expression of some inflammation-related genes has been shown to be correlated with a decreased hippocampal volume [12]. Moreover, several studies also demonstrated that the levels of inflammatory mediators are increased in the hippocampi of depression model rats subjected to chronic stress [13, 14]. Microglia, the resident immune cells of the central nervous system (CNS) [15], plays a central role in mediating the inflammatory response in MDD [16]. Mounting evidence indicates that microglia in several brain regions, especially the hippocampus, might be linked to the pathogenesis of depression in both depressed suicide cases and animal models [17–19]. The hippocampus comprises several subregions, including the cornu ammonis (CA) 1, CA2/3, and dentate gyrus (DG) subregions, and each subregion plays specific functional roles [20, 21]. Several studies have reported that the number of microglia in the CA2/3 and DG is increased in depression animal models exposed to chronic stress [17, 19, 22]. In contrast, Kreisel et al. reported that chronic unpredictable stress (CUS)-exposed mice exhibit decreased numbers of microglia in the DG of the hippocampus [23]. Thus, the types of changes in the number of microglia that occur in the hippocampal subregions in depression models are still controversial. To further explore this issue, the current study used an unbiased stereological technique to precisely quantify microglia in specific subregions of the hippocampus (the CA1, CA2/3, and DG) in depression model rats.

Microglia are characterized by different states, including a quiescent state in which the cells are ramified or resting and an activated state in which the cells are hyper-ramified or phagocytic [24]. In their quiescent state, ramified microglia have been found to monitor synaptic function and actively respond to neuronal activity [25]. Once brain homeostasis is disturbed, microglia integrate inflammatory signals in the periphery and the CNS and enter an activated state through structural and functional changes [26]. The limitations of the morphological classification standards of microglia in different states have promoted more functional research. A neuroimaging study demonstrated significant elevations in the density of translocator protein, a marker of microglial activation, in the hippocampi of patients currently experiencing a major depressive episode [27]. Furthermore, recent preclinical studies have demonstrated that the activation of microglia and the release of proinflammatory cytokines can be induced by chronic stress and can increase vulnerability to depressive-like behaviors [28]. Therefore, activated microglia and concomitant dysregulated neuroinflammation in the hippocampus might play a vital role in the development of depression. However, the change in the number of activated microglia in the hippocampus that occur in the context of depression remain unclear. To further investigate the functional changes in microglia associated with depression, we analyzed microglial activation and concomitant inflammatory cytokine levels in the hippocampi of CUS-exposed rats.

Physical exercise is an effective nonpharmaceutical treatment for MDD. Various clinical surveys have demonstrated that exercise is effective in improving depressive symptoms in individuals with mild or moderate depression [29, 30]. Our previous rodent studies further revealed that moderate-intensity treadmill running exercise can improve depressive-like behaviors in rats exposed to CUS [31–33]. However, the mechanisms underlying the antidepressant effects of exercise in MDD are unclear. As mentioned above, hippocampal microglia and the inflammatory response are associated with the development of depression. An inhibitor of activated microglia, minocycline, has been shown to attenuate neuroinflammation induced by lipopolysaccharide or interferon-alpha and ameliorate depressive-like behavior [34, 35], implying that inhibiting microglial activation might be a potential therapeutic strategy for depression. Numerous clinical and preclinical studies have suggested that exercise can prevent or attenuate depression and that this effect is correlated with decreases in neuroimmune factor levels [36, 37]. In addition, running exercise has been shown to decrease the numbers of microglia in the hippocampi of high-fat diet (HFD)-fed model rats and aged mice [38, 39]. Kohman et al. found that exercise contributed to reducing the activation of microglia isolated from the hippocampi of aged mice [40]. However, the effects of running exercise on the numbers and activation of hippocampal microglia and the accompanying inflammatory response in depression still need to be explored.

Therefore, we hypothesized that running exercise attenuates depressive symptoms by exerting protective effects against microglial activity and inflammatory cytokines in CUS models. To test this hypothesis, we verified the antidepressant effects of running exercise with a sucrose preference test (SPT). Then we investigated whether running exercise affects these changes using immunohistochemistry and unbiased stereological methods. Finally, we investigated the effects of treadmill exercise on microglial activation and the levels of inflammatory cytokines using immunofluorescence assays, polymerase chain reaction (PCR), and enzyme-linked immunosorbent assay (ELISA).

## Materials and methods

### Animals

**Fig. 1:**
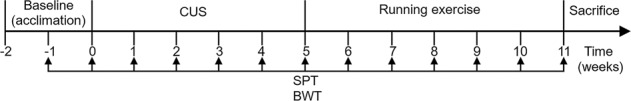
Timeline of the experimental procedures. The experiment lasted a total of 13 weeks. The animals were allowed to habituate to the housing conditions for 2 weeks before any interventions were initiated. The CUS model rats were exposed to two stressors per day for 5 weeks. Then the CUS + RUN group rats were subjected to treadmill running exercise for 6 weeks. Sucrose preference and body weight were assessed during a fixed time frame per week. CUS chronic unpredictable stress, CUS + RUN CUS + running, SPT sucrose preference test, BWT body weight test.

Sixty male Sprague-Dawley rats (6–8 weeks old and weighing 200 ± 20 g) were provided by the Experimental Animal Center of Chongqing Medical University (Chongqing, P. R. China). The rats were housed under a 12-h light/dark cycle at an appropriate temperature (22 ± 2 °C) and provided access to food and water. Before the interventions began, all rats were allowed to adapt to the housing conditions for 2 weeks. During the animal experiment, the animals in each group were treated by the investigators without blinding. All procedures (Fig. 1) were approved by the Animal Care Committee of Chongqing Medical University and performed in accordance with the National Institutes of Health Guide for the Care and Use of Laboratory Animals.

### CUS paradigm

The CUS procedure was performed as described in our previous reports [32, 41]. After a 2-week baseline adjustment of sucrose preference, rats with sucrose preference above baseline were randomly divided into the control group (*n* = 23) and the CUS group (*n* = 37). The control rats were housed at a density of four to five rats per cage, and the CUS rats were housed in individual cages. The two groups of rats were housed in separate rooms and had no contact with each other. The model rats were subjected to different types of stressors, including water or food deprivation, empty bottle exposure, intermittent illumination, cage tilting, restraint, damp bedding exposure, no bedding exposure, cold/hot stress, strobe light exposure, noise exposure, electric foot shock exposure, tail pinching, and cage shaking. Two types of stressors were applied daily, and no stimulus was repeated within 5 days (Table 1). The entire CUS procedure lasted for 5 weeks. Then the CUS rats were randomly divided into the CUS + standard group (CUS + STD group, *n* = 17) and the CUS + running group (CUS + RUN group, *n* = 20).

**Table 1:** Schedule of the CUS paradigm.

Time	Monday	Tuesday	Wednesday	Thursday	Friday	Saturday	Sunday
Week 1	Cage tilting	Water deprivation	Strobe light	Continuous lighting	Noise	Food deprivation	SPT
	Damp bedding	Continuous darkness during daytime	Electric foot shock	Restraint	Tail pinching	Intermittent illumination	BWT
Week 2	Cage tilting	Continuous lighting	Water deprivation	Empty bottle exposure	Cold stress	Intermittent illumination	SPT
	Damp bedding	Cage shaking	Electric foot shock	Continuous darkness during daytime	Strobe light	Restraint	BWT
Week 3	Food deprivation	Water deprivation	Strobe light	Cage tilting	Intermittent illumination	Continuous darkness during daytime	SPT
	Continuous darkness during daytime	Hot stress	Tail pinching	Bedding removed	Noise	Restraint	BWT
Week 4	Water deprivation	Empty bottle exposure	Cage tilting	Continuous lighting	Food deprivation	Intermittent illumination	SPT
	Restraint	Electric foot shock	Bedding removed	Cage shaking	Strobe light	Cold stress	BWT
Week 5	Continuous lighting	Water deprivation	Food deprivation	Continuous darkness during daytime	Cage tilting	Intermittent illumination	SPT
	Noise	Hot stress	Tail pinching	Restraint	Damp bedding	Cage shaking	BWT

*SPT* sucrose preference test, *BWT* body weight test.

### Treadmill running protocol

In the CUS + RUN group, the rats ran on a six-lane motorized treadmill for 20 min each day, 5 days per week for 6 weeks (Fig. 1). During the first week, the treadmill speed was gradually increased from 10 to 20 m/min. During the remaining 5 weeks, the speed was maintained at 20 m/min. This moderate-intensity treadmill running pattern was used as described in our previous study [32].

During the running exercise period, the rats in the CUS + STD group and the CUS + RUN group were housed at a density of one rat per cage, while the rats in the control group remained housed under normal circumstances with four to five rats per cage.

### Behavior tests

#### Body weight test

The body weights of the rats were measured during a fixed time frame each week.

#### Sucrose preference test

The SPT was carried out as previously described [42]. Before the experiment, the rats were acclimated to the sucrose solution (1%, w/v). Briefly, two bottles of sucrose solution were placed in each cage for 24 h and then replaced with two bottles filled with sucrose solution and water. After the acclimation period, the rats were allowed access to one bottle of sucrose solution and one bottle of water.

After 24 h, the weights of the consumed sucrose solution and water were recorded. The percentage of sucrose preference was calculated as sucrose consumption/(sucrose consumption + water consumption) × 100% [43]. The SPT was assessed during a fixed time frame each week.

### Perfusion and tissue processing

In the following procedures, all experiments and data analyses were carried out by investigators blinded to the treatment conditions. After the behavioral testing, five rats were randomly selected from each group. The rats were anesthetized with 1% pentobarbital sodium (4 mL/kg, intraperitoneal injection) and transcardially perfused with heparinized saline followed by 4% paraformaldehyde in phosphate-buffered saline (PBS, 0.6 M, pH 7.4). Then the cerebrum of each rat was removed and cut into two hemispheres along the sagittal suture. The brain tissue was postfixed in 4% paraformaldehyde for 24 h and subsequently immersed in a series of sucrose solutions of increasing concentration. The right or left hemisphere was chosen randomly and cut into 50 μm serial sections using a cryostat (CM1860, Leica, Germany). Every sixth section containing the hippocampus was sampled in a systematic random manner, and 12 sets of sampled sections were acquired for the following analyses.

### Immunohistochemistry and stereological analyses

Two separate sets of serial sections containing the hippocampus were chosen from each group of rats. The free-floating sections were rinsed in PBS with 0.3% Triton X-100 (Sigma, USA) and 0.1% Tween (PBS + T) 6 times for 10 min each. Then, the sections were blocked with 1% fetal bovine serum (FBS), 10% SP 9001-A, 0.1% cold water fish gelatin, and PBS + T for 2 h at 37 °C. The sections were then incubated with a rabbit anti-Iba1 primary antibody (1:2000, ab178847, Abcam, Cambridge, UK) in PBS + T for 2 days at 4 °C. After being rewarmed and rinsed, the sections were incubated with SP 9001-B (biotinylated goat anti-rabbit immunoglobulin, 1:20) for 3 h at 37 °C and followed by SP 9001-C (biotin-HRP-streptavidin) for 2 h at 37 °C. Subsequently, the sections were immersed in a diaminobenzidine solution (ZLL-9032, ZSGB, Beijing, P. R. China) for approximately 1 min. Finally, the sections were dehydrated by sequential immersion in a graded ethanol series (75, 95, and 100%; 5 min each) and xylene (3 × 10 min).

All quantitative analyses were performed as previously described [41, 44]. The hippocampal fields were delineated by cytoarchitectonic criteria. According to the morphological features of the neurons in the hippocampus, the contours of the CA1, CA2/3 and DG were delineated at a ×2.5 magnification (Supplementary Fig. 1A). The numbers of Iba1^+^ cells in the three subfields of the hippocampus were estimated using the optical fractionator method in the stereological system (Glostrup, Denmark). The total number of Iba1^+^ cells in each hippocampal subregion was calculated using the following formula:

$$N = {\sum} {Q^ - \times \frac{1}{{{\rm{ssf}}}} \times \frac{1}{{{{{\mathrm{asf}}}}}} \times \frac{1}{{{\rm{hsf}}}}}$$

where the variable *N* is the total number of Iba1^+^ cells in the CA1, CA2/3, and DG in each animal, ∑*Q*^−^ is the total number of Iba1^+^ cells actually counted in the specimens, ssf is the section sampling fraction, asf is the area sampling fraction, and hsf is the section height sampling fraction (for the stereological sampling scheme, see Supplementary Fig. 1B, C and Table 2). In the present study, the ssf was 1/6 according to the above section sampling method. The optical disector counting frames were placed in the delineated area of the sections in a systematically random manner. The asf, the ratio between the area of the unbiased counting frame and the rectangular area, was set to 6%. The guard zone was set at a thickness of 3 μm, and microglia were counted at a depth of 15 μm below the guard zone. Hsf represents the ratio between the counted height and the mean thickness of each section.

**Table 2:** Sampling scheme for the estimation of the number of Iba1^+^ cell.

	Control group	CUS + STD group	CUS + RUN group
Number of sections sampled			
CA1	13–17	13–18	13–18
CA2/3	12–17	12–17	13–18
DG	14–20	14–19	14–19
Number of counting frames sampled			
CA1	260 (244–298)	343 (310–378)	288 (230–334)
CA2/3	214 (176–273)	269 (237–293)	238 (193–309)
DG	262 (204–345)	306 (271–352)	301 (235–350)
Number of Iba1^+^ cells counted			
CA1	391 (274–462)	650 (459–804)	436 (253–548)
CA2/3	317 (197–517)	520 (368–747)	359 (197–514)
DG	439 (263–598)	637 (471–818)	502 (301–702)

Note: The number of sampled sections is presented as the range, whereas the numbers of counting frames and the numbers of sampled Iba1^+^ cells are presented as the mean with the range in parentheses.

*CUS* *+* *STD* CUS + standard, *CUS* *+* *RUN* CUS + running.

### Coimmunostaining and cell counting

One set of serial sections containing the hippocampus was randomly chosen from each group of rats. Free-floating sections were rinsed with PBS + T 6 times for 10 min each. Then the sections were blocked with 1% FBS, 10% SP 9001-A, and PBS + T for 2 h at 37 °C. The sections were then incubated with a rabbit anti-Iba1 primary antibody (1:500, ab178847, Abcam, Cambridge, UK) and a mouse anti-CD68 primary antibody (1:100, ab955, Abcam, Cambridge, UK) in PBS + T for 2 days at 4 °C. The sections were washed three times for 10 min at room temperature in PBS + T. For the immunofluorescence staining, the sections were incubated with DyLight 549- and DyLight 488-labeled secondary antibodies (1:200; Abbkine, USA) for 2 h at 37 °C. 4’,6-Diamidino-2-phenylindole (AR1176, Boster, Wuhan, P. R. China) was used for the nuclear staining. Fluorescent images were captured at a ×200 magnification under a Zeiss fluorescence microscope (Zeiss, Germany). At least ten representative images of each hippocampal subregion were acquired from each rat. The Iba1^+^/CD68^+^ cells in each image were manually quantified, and the cell density and percentage were calculated and analyzed. The percentage of activated microglia is expressed as the ratio of Iba1^+^/CD68^+^ cells to Iba1^+^ cells.

### Quantitative real-time PCR (qRT-PCR)

The levels of the proinflammatory cytokines IL-1β, inducible nitric oxide synthase (iNOS), IL-6, and tumor necrosis factor (TNF)-α are associated with the severity of depression [7, 45]. To evaluate the mRNA levels of these proinflammatory cytokines in the hippocampi of depression model rats, we randomly selected five rats from each group for the qRT-PCR analysis. The brain tissues of the rats were quickly removed on ice after anesthesia and decapitation. The hippocampal tissues were dissected, frozen in liquid nitrogen, and subsequently stored at −80 °C.

The total RNA was extracted from the hippocampi of the rats using a Total RNA Extraction Kit (LS1040, Promega, Shanghai, P. R. China) according to the manufacturer’s protocol. Complementary DNA (cDNA) synthesis was performed using a PrimeScript RT Reagent Kit with gDNA Eraser (RR047A, Takara, Japan) according to the manufacturer’s instructions. The primer sequences and sequence specificity of the primers were designed and tested, respectively, using the National Center for Biotechnology Information (NCBI) Primer-BLAST. The primer sequences are listed in Table 3. PCR amplification of cDNA was performed using 2× SYBR Green qPCR Master Mix (B21202, Bimake, Houston, USA). The formation of PCR products was detected in real time using a CFX96 Real-Time PCR Detection System (Bio-Rad, Hercules, CA, USA). A melting curve analysis was used to verify the primer specificity. Finally, the relative gene expression was analyzed by the 2^−ΔΔCt^ method.

**Table 3:** Primer sequences for qRT-PCR.

Gene	Forward primer (5′– 3′)	Reverse primer (5′– 3′)
IL-1β	TGAGGCTGACAGACCCCAAAAGAT	GCTCCACGGGCAAGACATAGGTAG
iNOS	GAGACGCACAGGCAGAGGTTG	AGCAGGCACACGCAATGATGG
IL-6	ACTTCCAGCCAGTTGCCTTCTTG	TGGTCTGTTGTGGGTGGTATCCTC
TNF-α	TCGTAGCAAACCACCAAGCG	AGAGAACGGATGAACACGCCA
β-Actin	TGTCACCAACTGGGACGATA	GGGGTGTTGAAGGTCTCAAA

*qRT-PCR* quantitative real-time polymerase chain reaction.

### Enzyme-linked immunosorbent assay

The protein expression levels of IL-1β, iNOS, IL-6, and TNF-α in the hippocampus were examined by ELISA. The hippocampi of the rats were isolated on ice and washed with normal saline. Then the tissue samples were homogenized and centrifuged at 12,000 rpm for 15 min. The supernatant was collected, and the total protein concentration was determined using a BCA Protein Assay Kit (Beyotime, P. R. China). Samples containing equivalent amounts of protein were calculated and subpackaged. Subsequently, the protein concentrations of inflammatory cytokines in the hippocampus were determined using ELISA kits (EIAab, P. R. China) according to the manufacturer’s instructions. The optical density at 450 nm was determined using a microplate reader (Bio-Rad, Hercules, CA, USA), and the concentration of these cytokines was calculated according to standard curves.

## Statistics

The statistical analyses were performed using SPSS version 23.0 (IBM, Armonk, NY, USA). All data are expressed as the mean ± standard deviation (SD). All data were assessed for normality and homogeneity of variance. The body weight data were analyzed using a repeated-measures analysis of variance (ANOVA). Regarding the remaining data, one-way ANOVA followed by a least significant difference post hoc test was used to compare the results among multiple groups and Student’s *t* tests were used for comparisons between two groups. The sample size of each experiment was chosen based on previous experience with the aim of detecting at least a *p* value <0.05 in the different tests applied. No animals were excluded from the present study.

## Results

### Running exercise reversed depressive-like behavior in CUS-exposed rats

**Fig. 2:**
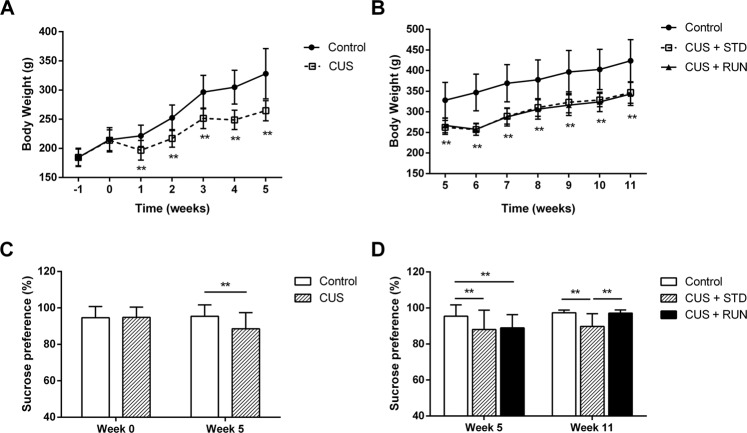
The effects of running exercise on the depressive-like behaviors of CUS-exposed rats. **A** Body weights of the rats in the control group (*n* = 23) and the CUS group (n = 37) during the first 7 weeks. **B** Body weights of the rats in the control group (*n* = 23), the CUS + STD group (*n* = 17) and the CUS + RUN group (*n* = 20) during the last 7 weeks. **C** Sucrose preferences of the rats in the control group (*n* = 23) and the CUS group (*n* = 37) at different stages. **D** Sucrose preferences of the rats in the control group (*n* = 23), the CUS + STD group (*n* = 17), and the CUS + RUN group (*n* = 20) at different stages. **p < 0.01. CUS + STD CUS + standard, CUS + RUN CUS + running.

The effect of running exercise on CUS-induced depressive-like behavior was analyzed by behavioral tests. The body weight of the CUS group was similar to that of the control group during the 2-week baseline adjustment phase (*p* > 0.05, Fig. 2A). The body weight of the CUS-exposed rats was found to be significantly lower than that of the normal rats from the first week of the CUS intervention (*p* < 0.01, Fig. 2A). During the running exercise period, the rats in the CUS + STD group and CUS + RUN group persistently showed distinctly lower body weights than the rats in the control group (*p* < 0.01, *p* < 0.01, respectively; Fig. 2B). In addition, the sucrose consumption of the normal rats was similar to that of the CUS-exposed rats during the baseline period (*t* = −0.170, *p* = 0.866, Fig. 2C). However, after the 5-week CUS intervention, the sucrose preference in the CUS group was significantly lower than that in the control group (*t* = −4.990, *p* = 0.000, Fig. 2C). After 6 weeks of running exercise, there were significant differences in sucrose preference among the three groups (*F*_(2,57)_ = 28.003, *p* = 0.000). The rats in the CUS + RUN group showed a significantly higher sucrose preference than the rats in the CUS + STD group (*p* = 0.000, Fig. 2D).

### Running exercise decreased the numbers of microglia in subfields of the hippocampus in the CUS-exposed rats

**Fig. 3:**
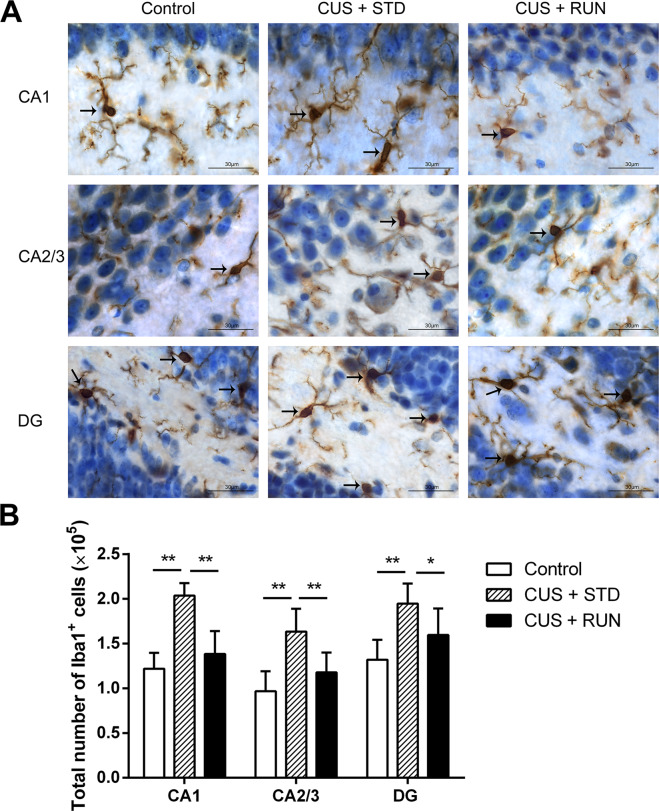
The effects of running exercise on the numbers of microglia in subfields of the hippocampus in CUS-exposed rats. **A** Representative images of immunohistochemical staining of Iba1 in each subregion of the hippocampus (×100 oil). Scale bar = 30 μm. **B** Quantitative analyses comparing the numbers of Iba1^+^ cells in the CA1, CA2/3, and DG areas among the control group, the CUS + STD group, and the CUS + RUN group. The data are expressed as the mean ± SD (*n* = 5 per group). *p < 0.05 and **p < 0.01. CUS + STD CUS + standard, CUS + RUN CUS + running.

The number of Iba1^+^ microglia in the hippocampus was observed and quantified using a stereological method combined with immunohistochemistry. Representative pictures of the immunohistochemical staining using an anti-Iba1 antibody are shown in Fig. 3A. Iba1 is a protein specifically expressed in microglia in the CNS. There were significant differences in Iba1^+^ microglia in the CA1, CA2/3, and DG among the three groups (*F*_(2,12)_ = 24.182, *p* = 0.000; *F*_(2,12)_ = 10.510, *p* = 0.002; *F*_(2,12)_ = 7.899, *p* = 0.006). Specifically, there were markedly more Iba1^+^ microglia in the CA1, CA2/3, and DG in the CUS + STD group of rats than in the control group of rats (*p* = 0.000, *p* = 0.001, *p* = 0.002, respectively). Moreover, after 6 weeks of running exercise, the CUS + RUN group had decreased numbers of Iba1^+^ microglia in the CA1, CA2/3, and DG compared with the CUS + STD group (*p* = 0.000, *p* = 0.010, *p* = 0.047, respectively; Fig. 3B and Table 4). In the present study, sampling met the criteria because the observed variance of the individual estimate (OCE^2^) was much <50% of the observed interindividual variance (OCV^2^) (Table 4).

**Table 4:** Stereological results of the total numbers of Iba1^+^ cells in the hippocampus.

	Control group	CUS + STD group	CUS + RUN group
CA1			
Number of Iba1^+^ cells (×10^5^)	1.22 ± 0.18	2.04 ± 0.14**	1.38 ± 0.26^##^
OCV (×10^−3^)	144.97	68.14	185.10
OCE (×10^−3^)	2.89	2.22	2.73
CA2/3			
Number of Iba1^+^ cells (×10^5^)	0.97 ± 0.22	1.63 ± 0.26**	1.18 ± 0.22^##^
OCV (×10^−3^)	231.96	157.34	189.71
OCE (×10^−3^)	3.28	2.50	2.97
DG			
Number of Iba1^+^ cells (×10^5^)	1.32 ± 0.22	1.95 ± 0.23**	1.60 ± 0.29^#^
OCV (×10^−3^)	167.96	115.85	184.56
OCE (×10^−3^)	2.79	2.28	2.54

Note: The numbers of Iba1^+^ cells are represented as the mean ± SD.

*CUS* *+* *STD* CUS + standard, *CUS* *+* *RUN* CUS + running, *OCE* observed coefficient of error, *OCV* observed coefficient of variation.

**p < 0.01 when the CUS + STD group was compared with the control group. ^#^*p* < 0.05 when the CUS + RUN group was compared with the CUS + STD group. ^##^*p* < 0.01 when the CUS + RUN group was compared with the CUS + STD group.

### Running exercise decreased the density and ratio of activated microglia in subfields of the hippocampus in the CUS-exposed rats

**Fig. 4:**
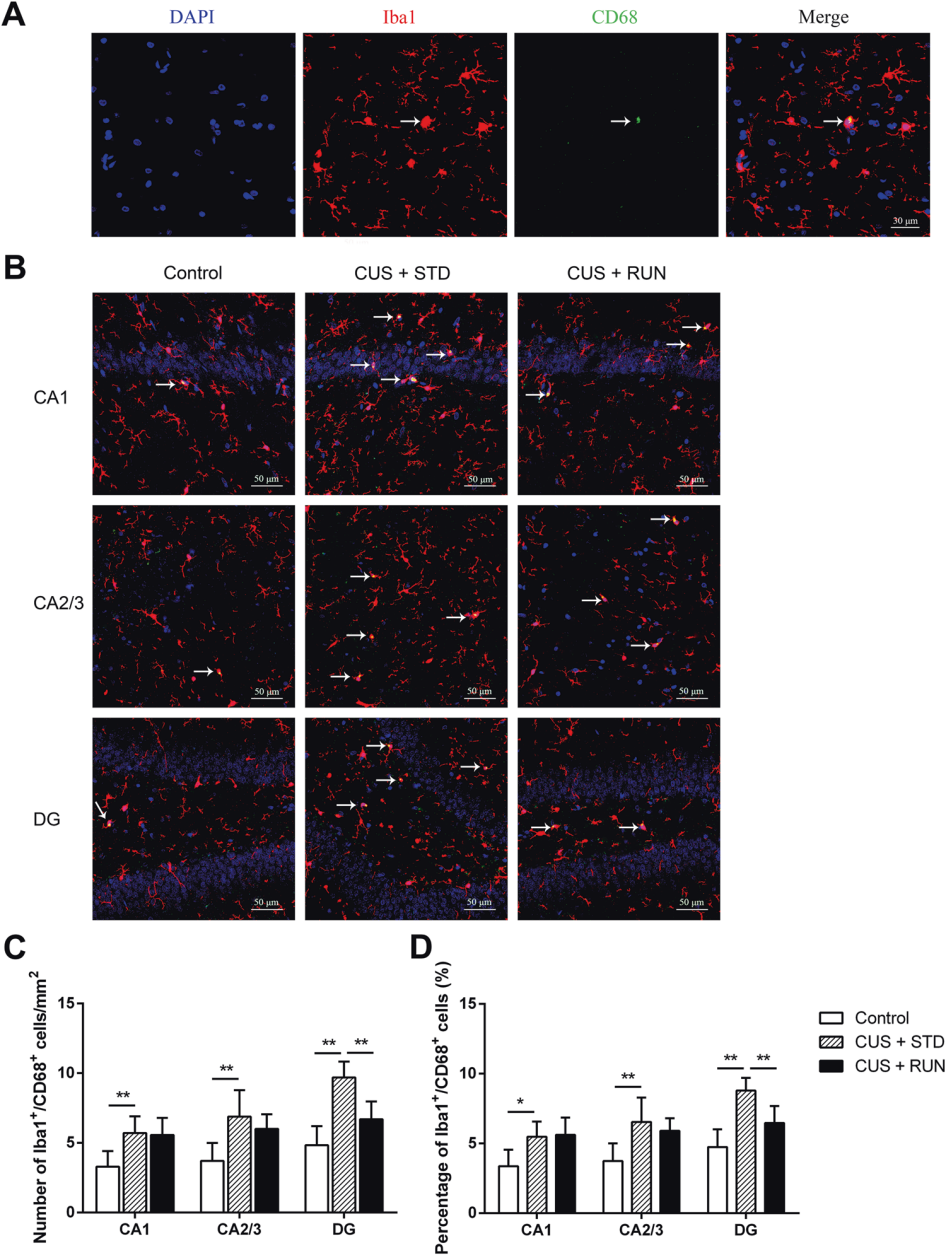
The effects of running exercise on the density and ratio of activated microglia in subfields of the hippocampus in CUS-exposed rats. **A** Representative confocal images of Iba1^+^/CD68^+^ microglia in the hippocampus. Scale bar = 30 μm. **B** Representative confocal images of immunofluorescence staining using antibodies against Iba1 and CD68 in the hippocampal subregions in the control group, the CUS + STD group, and the CUS + RUN group. The arrowheads indicate representative Iba1^+^/CD68^+^ cells. Scale bar = 50 μm. **C**, **D** Quantitative analyses comparing the densities and percentages of Iba1^+^/CD68^+^ cells in the CA1, CA2/3, and DG areas among the control group, the CUS + STD group, and the CUS + RUN group. The data are expressed as mean ± SD (n = 5 per group). *p < 0.05 and **p < 0.01. CUS + STD CUS + standard, CUS + RUN CUS + running.

The activation status of microglia was observed using Iba1 and CD68 immunofluorescence staining. CD68 is a transmembrane glycoprotein that is highly expressed in activated and phagocytic microglia. Red staining indicated Iba1^+^ microglia, and green staining indicated CD68 (Fig. 4A, B). There were significant differences in the densities of Iba1^+^/CD68 ^+^ microglia in the CA1, CA2/3, and DG (*F*_(2,12)_ = 6.482, *p* = 0.012; *F*_(2,12)_ = 6.349, *p* = 0.013; *F*_(2,12)_ = 18.676, *p* = 0.000) and the percentages of Iba1^+^/CD68^+^ microglia in Iba1^+^ cells in the CA1, CA2/3, and DG (F_(2,12)_ = 5.851, *p* = 0.017; *F*_(2,12)_ = 5.935, *p* = 0.016; *F*_(2,12)_ = 15.876, *p* = 0.000) among the three groups. Specifically, relative to the rats in the control group, the densities of Iba1^+^/CD68^+^ microglia and percentages of Iba1^+^/CD68^+^ microglia in Iba1^+^ cells in the CA1, CA2/3, and DG areas were elevated in the CUS + STD group rats (*p* = 0.008, *p* = 0.005, and *p* = 0.000 for the densities of Iba1^+^/CD68 ^+^ cells; *p* = 0.014, *p* = 0.006, and *p* = 0.000 for the percentages of Iba1^+^/CD68^+^ cells), indicating that CUS promoted microglial activation. In addition, the density of Iba1^+^/CD68^+^ microglia and percentage of Iba1^+^/CD68^+^ microglia in Iba1^+^ cells in the DG area of the hippocampus in the CUS + RUN group were found to be decreased (*p* = 0.003 and *p* = 0.007, respectively). However, the densities of Iba1^+^/CD68^+^ microglia and percentages of Iba1^+^/CD68^+^ microglia in Iba1^+^ cells in CA1 and CA2/3 of the hippocampus in the CUS + RUN group did not significantly differ from those in the CUS + STD group (*p* = 0.857 and *p* = 0.36 for the density of Iba1^+^/CD68^+^ cells; *p* = 0.863 and *p* = 0.463 for the percentage of Iba1^+^/CD68^+^ cells) (Fig. 4C, D and Table 5).

**Table 5:** Density and percentage of Iba1^+^/CD68^+^ cells.

	Control group	CUS + STD group	CUS + RUN group
Density of Iba1^+^/CD68 ^+ ^cells (/mm^2^)			
CA1	3.30 ± 1.12	5.70 ± 1.21**	5.56 ± 1.23
CA2/3	3.71 ± 1.28	6.89 ± 1.90**	6.01 ± 1.05
DG	4.83 ± 1.37	9.69 ± 1.14**	6.69 ± 1.29^##^
Percentage of Iba1^+^/CD68^+^ cells (%)			
CA1	3.37 ± 1.18	5.49 ± 1.08*	5.62 ± 1.23
CA2/3	3.75 ± 1.26	6.55 ± 1.74**	5.90 ± 0.90
DG	4.75 ± 1.27	8.80 ± 0.91**	6.47 ± 1.21^##^

Note: The density and percentage of Iba1^+^ cells are presented as mean ± SD.

*CUS* *+* *STD* CUS + standard, *CUS* *+* *RUN* CUS + running.

*p < 0.05 when the CUS + STD group was compared with the control group. **p < 0.01 when the CUS + STD group was compared with the control group. ^##^*p* < 0.01 when the CUS + RUN group was compared with the CUS + STD group.

### Running exercise reversed the increases in the mRNA levels of inflammatory cytokines in the hippocampus of the CUS-exposed rats

**Fig. 5:**
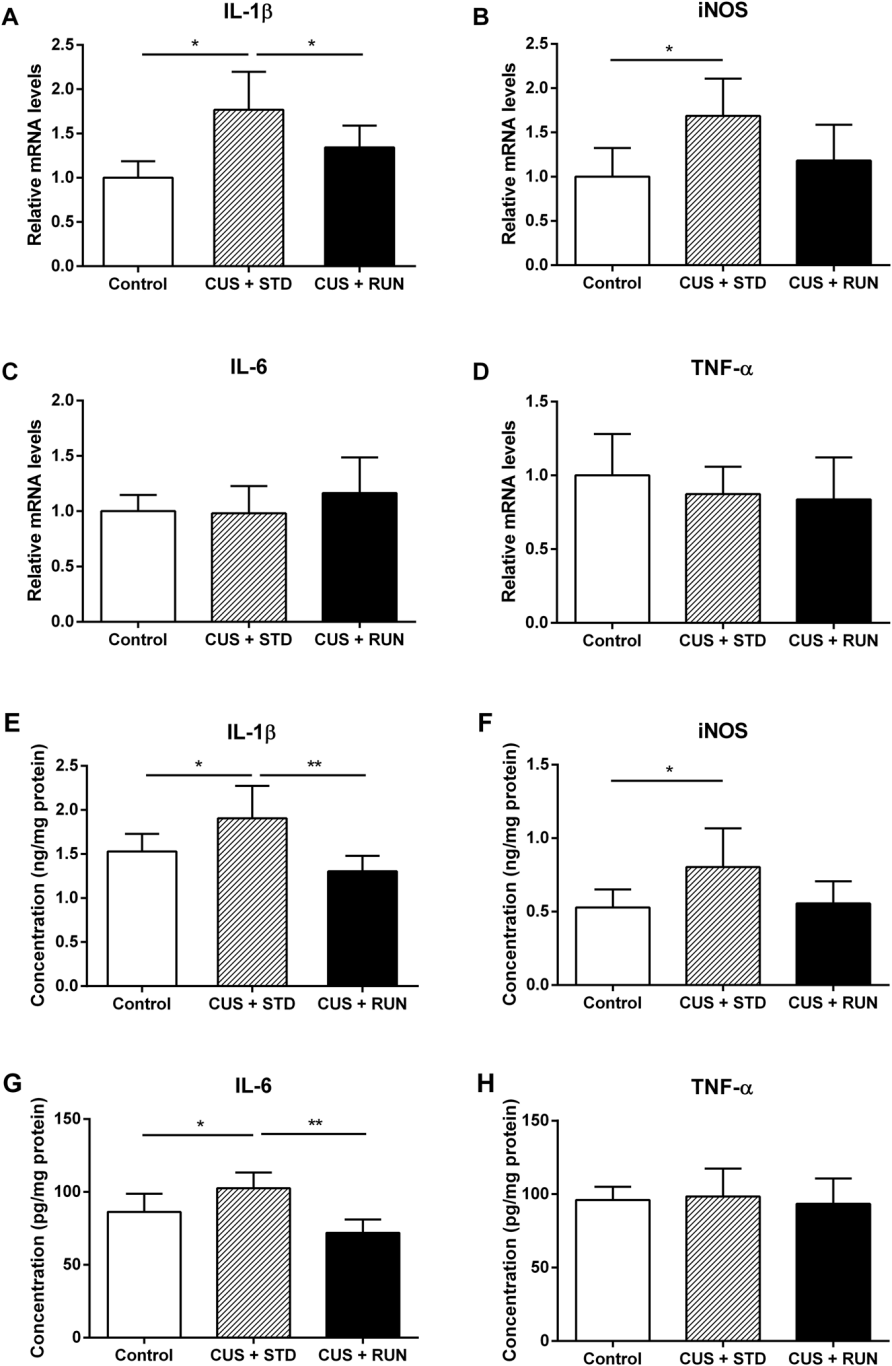
The effects of running exercise on the mRNA and protein levels of proinflammatory cytokines in the hippocampus in CUS-exposed rats. **A**–**D** Quantitation of the relative mRNA expression of the proinflammatory cytokines IL-1β, iNOS, IL-6, and TNF-α in the hippocampi of the control group, the CUS + STD group, and the CUS + RUN group. The data are presented as mean ± SD (*n* = 5 per group). **E**–**H** Quantitation of the protein levels of the proinflammatory cytokines IL-1β, iNOS, IL-6, and TNF-α in the hippocampi of the three groups. The data are presented as mean ± SD (*n* = 5 per group). *p < 0.05 and **p < 0.01. CUS + STD CUS + standard, CUS + RUN CUS + running.

The changes in inflammatory cytokines (IL-1β, iNOS, IL-6, and TNF-α) at the genetic level were detected by qRT-PCR. As shown in Fig. 5, there were significant differences in the mRNA levels of IL-1β and iNOS in the hippocampus among the three groups (*F*_(2,12)_ = 7.940, *p* = 0.006; *F*_(2,12)_ = 4.248, *p* = 0.040). Specifically, compared to the control group, the CUS exposure significantly increased the mRNA levels of IL-1β and iNOS in the hippocampus (*p* = 0.002 and *p* = 0.016, respectively; Fig. 5A, B). In addition, running exercise significantly decreased the mRNA levels of IL-1β in the hippocampi of the rats in the CUS + STD group (*p* = 0.048; Fig. 5A). There were no significant differences in the mRNA levels of IL-6 and TNF-α in the hippocampus among the three groups (*F*_(2,12)_ = 0.810, *p* = 0.468; *F*_(2,12)_ = 0.497, *p* = 0.621; Fig. 5C, D).

### Running exercise reversed the increases in the protein levels of inflammatory cytokines in the hippocampus of the CUS-exposed rats

The protein levels of inflammatory cytokines (IL-1β, iNOS, IL-6, and TNF-α) were measured by ELISA. There were significant differences in the protein levels of IL-1β and IL-6 in the hippocampus among the three groups (F_(2,12)_ = 6.725, *p* = 0.011; F_(2,12)_ = 10.003, *p* = 0.003; Fig. 5E, G). Specifically, the protein levels of IL-1β and IL-6 were enhanced in the CUS + STD group compared with those in the control group (*p* = 0.043 and *p* = 0.035, respectively). Compared with the CUS + STD group, the protein levels of IL-1β and IL-6 were significantly diminished in the hippocampi of the rats in the CUS + RUN group (*p* = 0.003 and *p* = 0.001, respectively). In addition, the protein levels of iNOS in the CUS + STD group were significantly increased compared to those in the control group (*p* = 0.040; Fig. 5F). However, the protein levels of TNF-α in the hippocampus did not significantly differ among the three groups (*F*_(2,12)_ = 0.136, *p* = 0.875; Fig. 5H).

## Discussion

In the present study, CUS was used to induce depressive-like behavior in rats. The CUS model can simulate the various stresses that humans encounter in life, causing rodents to exhibit the core symptoms of depression, such as anhedonia [46]. The SPT was performed to evaluate the degree of anhedonia of the CUS model rats. Consistent with a previous study [47], we found that 5 weeks of the CUS intervention reduced body weight and sucrose preference in the rats, indicating that the rat depression model was successfully established.

Increasing evidence suggests that alterations in the number of microglia are associated with the onset of depression, and the change in the number of total microglia helps determine whether microglia are in the activated or senescent phase [26, 48]. Iba1 is constitutively expressed in all microglia, including resting ramified microglia and activated microglia [49]. Thus, an analysis of Iba1^+^ microglia along with an assessment of depressive symptoms might aid in the prediction of the progression and treatment effects of depression. The hippocampus is a stress-sensitive brain area that has been implicated in the pathophysiology and progression of depression [50–53]. In the current study, our results show that the total number of Iba1^+^ microglia in the hippocampus in the depressed model rats was significantly higher than that in the control rats, suggesting that the total number of microglia increases in response to CUS exposure. In contrast, Cai et al. and Tong et al. examined the change in the number of microglia in a defined area of the hippocampus in CUS-exposed mice by an immunofluorescence assay and found that the density of microglia was decreased in the hippocampi of depressed mice [54, 55]. The first possible reason for the different effects of CUS on the number of microglia might be the differential quantitative methods used. In the studies by Cai et al. and Tong et al., the density of Iba1^+^ microglia in the DG of the hippocampus was evaluated via a semiquantitative measurement of Iba1^+^ material; however, the microglial density may not directly reflect the total number of microglia. In our study, an unbiased stereological technique, a three-dimensional quantitative method, was used to accurately quantify microglia in the hippocampus; thus, we provided more precise results. The second possible reason for the different observed effects of CUS on the number of microglia might be the types and duration of stressors. Cai et al. and Tong et al. established the CUS model by adopting ten different types of stressors. In our study, more types of stressors, including food deprivation, empty bottle exposure, no bedding exposure, hot stress, electric foot shock exposure, and tail pinching, were added to the CUS paradigm. The duration of each stress was generally different. We hypothesize that the heterogeneity of these stressors may be another important reason for the different changes in the number of microglia in animal models of depression. The third possible reason for the different observed effects of CUS on the number of microglia might be the different animal species studied. In the present study, which used a rat model, we found that the number of Iba1^+^ microglia was increased in the hippocampi of the depression model rats. This result is consistent with the findings of previous research in which a rat model was adopted [17]. Cai et al. and Tong et al. evaluated the changes in microglia using mouse models and found a decreased microglial density in the hippocampus. We hypothesize that hippocampal microglia in rats are more susceptible to CUS exposure than those in mice. In addition, we systemically measured the number of microglia in the hippocampal CA1, CA2/3, and DG. We found that CUS significantly increased the total number of Iba1^+^ microglia in each subregion of the hippocampus in the depression model rats and that the trends in the variation in the number of microglia across the three regions of the hippocampus after the CUS intervention were consistent. Therefore, we speculate that the changes in the total number of microglia (including activated and resting microglia) induced by CUS are consistent across the three subregions of the hippocampus. Collectively, our present results suggest that microglial number changes in the hippocampus may be involved in the pathogenesis of the depression model, and we are the first to provide evidence that CUS affects the number of microglia in each subregion of the hippocampus. Although most previous studies and the present study found microglial changes in the hippocampi of depression models [19, 22, 56, 57], changes in microglia in other brain regions cannot be ruled out, which merits further study.

In addition to the changes in the total number of microglia, microglial functional alterations, including microglial activation and concomitant elevations in pro-inflammatory biomarker levels, play crucial roles in the deterioration of depression [58, 59]. Changes in microglial activation state have been shown to be sensitive markers in the brains of depressed suicides [18]. Furthermore, numerous studies have reported that the levels of CD68, an ideal marker of activated and phagocytic microglia [60], are elevated in depression models [61–63]. Another study demonstrated that chronic stress increases the density of activated/phagocytic microglia in the DG of the hippocampus [64]. However, a systematic study on the change in activated microglia in each subregion of the hippocampus has not been reported. In the present study, we assessed microglial activation mainly by analyzing the number of microglia labeled by Iba1 and CD68 per unit area and found that CUS increased the density of Iba1^+^/CD68^+^ cells and the percentage of Iba1^+^/CD68^+^ cells among Iba1^+^ cells in the CA1, CA2/3, and DG of the hippocampus, revealing that chronic stress induced microglial activation in the hippocampus. Microglia can be activated during inflammatory conditions that cause deviations from microglial homeostasis, such as stress, stroke, infection, and neurodegenerative diseases [26]. In the current study, CUS exposure might be the reason for the increased microglial activation. However, many stress-related factors, such as stress hormones, neurotransmitters, and pattern recognition receptor agonists, can greatly affect the activation of microglia [65]. Thus, the specific mechanisms underlying stress-associated microglial activation need to be further studied. The current study investigated microglial activation in each subfield of the hippocampus in depression model rats from a functional perspective, and the results indicate that activated microglia in the hippocampus are involved in the pathogenesis of depression. Furthermore, we found that CUS induced elevated mRNA and protein levels of the proinflammatory cytokines IL-1β and iNOS in the hippocampus, which is consistent with a report by Liu et al. [45]. As IL-1β and iNOS released by microglia help amplify neuroinflammation in depression and subsequently lead to neurotoxicity and pathological changes [66, 67], this finding suggests that IL-1β and iNOS in the hippocampus may play an important role in the processes of microglial activation. However, no significant changes in the hippocampal gene expression of IL-6 were found in the depressed model rats. In contrast, the IL-6 protein level in the hippocampi of the CUS + STD group rats was significantly higher than that in the hippocampi of the control group rats. Gene expression is a complex process involving transcription, RNA splicing, translation, and posttranslational protein modification. The different results of the gene expression and protein level of IL-6 could be due to improved gene translation efficiency, enhanced posttranslational modifications, or mRNA instability, which ultimately affects the protein level, but the specific mechanism remains unclear and requires further study. Collectively, the evidence indicates that microglial activation and elevations in inflammatory cytokines may contribute to the development of depression.

Running exercise is recognized as an effective therapeutic strategy for depression [68]. Consistent with the results of a previous study [69], our behavioral results demonstrate that 6 weeks of running exercise increased the sucrose preference of the depression model rats, indicating that running exercise can mitigate the depressive-like behaviors induced by CUS. Therefore, these results confirm that running exercise has a therapeutic effect against depression. Zheng et al. showed that the inhibition of microglial activation helps attenuate IFN-α-induced neuroinflammation and depressive-like behaviors in mice [35], which may be associated with important antidepressant mechanisms. Several studies in related fields clearly demonstrated that treadmill exercise attenuates the increase in microglia in the hippocampal CA1 region and cerebellar vermis in subjects with neurodegenerative disorders, such as sporadic Alzheimer’s disease and Parkinson’s disease [70, 71]. However, whether running exercise has positive effects on hippocampal microglia in depression model rats has remained unclear. Using an unbiased stereological method, we found that running exercise could reverse the CUS-induced increases in Iba1^+^ microglial populations in the hippocampal CA1, CA2/3, and DG subregions in depression model rats. The current study is the first to provide unbiased and accurate quantitative evidence suggesting that the total number of microglia in the hippocampus is altered in CUS model rats after running exercise. More importantly, our results indicate that hippocampal microglia might be structural targets for the ameliorative effects of running exercise on depression.

As mentioned above, microglial activation and the levels of inflammatory factors can better reflect the effects of microglia on the neuroinflammatory response than the total number of microglia. In the present study, we found that running exercise attenuated the increases in the number of Iba1^+^ microglia in the CA1, CA2/3, and DG subregions of the hippocampus in the depression model rats but attenuated the increase in the density of Iba1^+^/CD68^+^ microglia only in the DG region of the hippocampus. Impaired functional activity in the hippocampal CA1, CA2/3, and DG subregions has been found to be associated with depressive symptoms [72], and the subregions of the hippocampus interact to modulate depressive emotions. The DG region acts as a gateway that processes sensory inputs from the environment and integrates these inputs into the hippocampus to moderate neuroplasticity [73]. A study by Wohleb et al. demonstrated that microglia mediate neuronal remodeling and synaptic deficits in depression model mice, indicating that neuronal plasticity is closely related to microglia during the development of depression [74]. In addition, treadmill exercise has been shown to have neuroprotective effects in the DG area of the hippocampus by inhibiting HFD-induced microglial activation and the expression of proinflammatory cytokines [75]. Intriguingly, running exercise can regulate hippocampal synaptic plasticity and neurogenesis by selectively enhancing long-term potentiation in the DG region instead of the CA1 [76]. Thus, we speculate that activated microglia in the DG area are more likely to respond to running exercise treatment than those in other hippocampal subregions in the context of depression. Moreover, as the sensitivity of the microglial state might vary across different hippocampal regions, we speculate that CUS-induced activated/phagocytic microglia in the CA1 and CA2/3 of the hippocampus might not transition to the quiescent state or might not transition as quickly as microglia in the DG region after running exercise. The regional specificity of running exercise in alleviating microglial activation and neuroinflammation needs to be further investigated in future studies. In addition, it can be concluded that physical exercise reduces the detrimental effects of neuroinflammation in MDD patients and depression model rodents [77, 78]. Our results show that running exercise reduced the gene expression and protein level of IL-1β in the hippocampus of rats subjected to CUS. Furthermore, running exercise reduced the protein level of IL-6 in the hippocampus of the depression model rats. There were no differences in the gene expression and protein levels of iNOS and TNF-α after running wheel exercise. Finally, our results prove that running exercise has protective effects against the elevation of some inflammatory factors in the hippocampus of CUS model rats. Taken together, we speculate that running exercise has antidepressant effects by suppressing microglial activation and the release of inflammatory cytokines.

In conclusion, our study shows that the number and activation of hippocampal microglia along with accompanying neuroinflammation in the hippocampus may be involved in the pathogenesis of depression in model rats. In addition, running exercise reverses these changes in the context of depression, indicating that changes in hippocampal microglia and neuroinflammation might be the structural bases by which running exercise exerts antidepressant effects. Our findings may shed new light on immunomodulatory targets related to the antidepressant effects of exercise and provide a scientific basis for the clinical treatment strategy for depression.

## Supplementary information

Figure legends of supplementary Figure 1

Supplementary figure 1
