# Supplementary material for: Beneficial effects of running exercise on hippocampal microglia and neuroinflammation in chronic unpredictable stress-induced depression model rats
Source: Transl Psychiatry. 2021 Sep 6;11:461. doi: 10.1038/s41398-021-01571-9 (PMC8421357; doi:10.1038/s41398-021-01571-9)
Supplement: Supplementary file 1 — Figure legends of supplementary Figure 1 [file 41398_2021_1571_MOESM1_ESM.docx]

# Supplementary Fig. 1


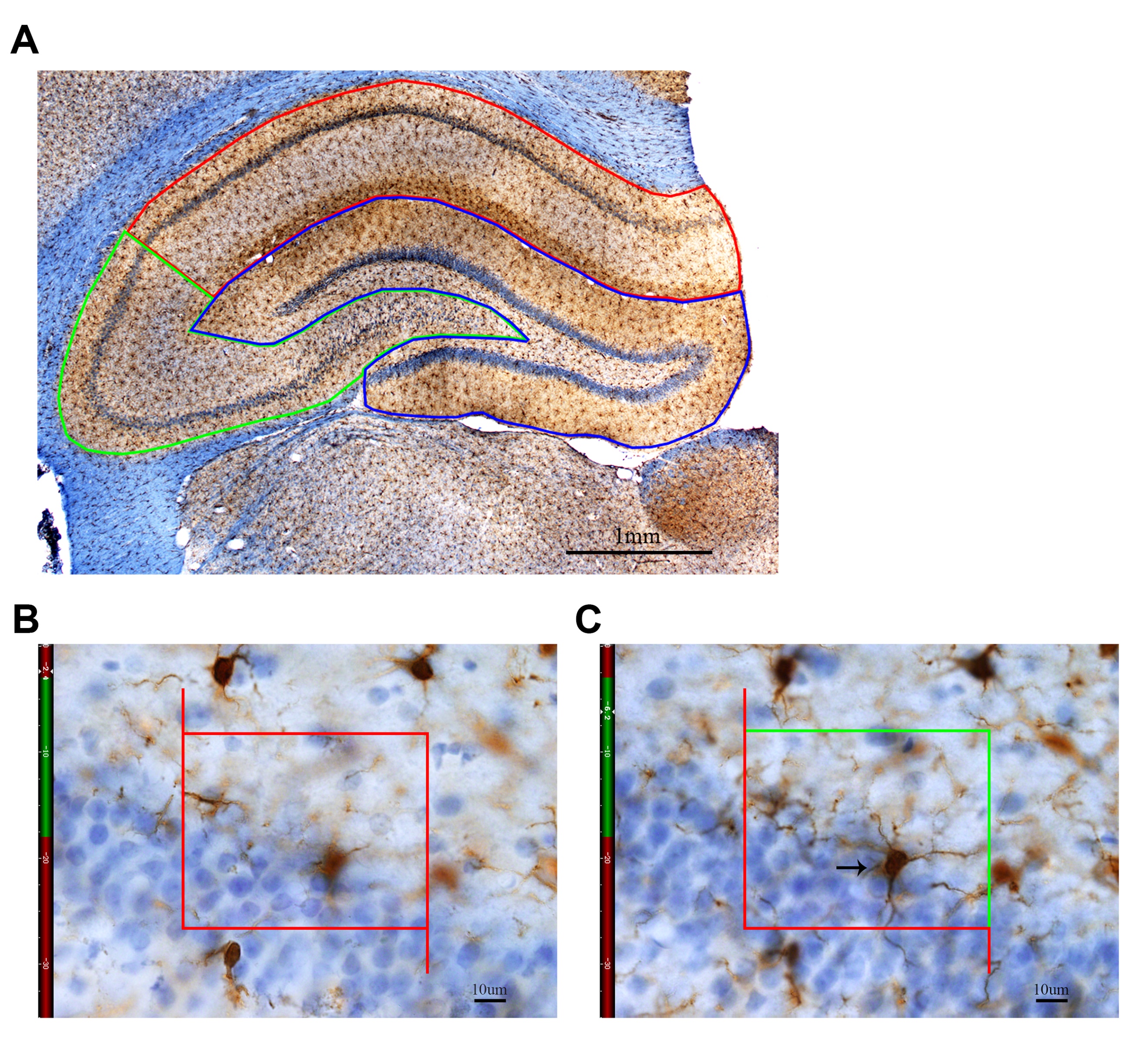


**Supplementary Fig. 1. Illustrations of hippocampal subregions and the unbiased counting method. (A)** Representative outlines of different hippocampal subregions. The CA1, CA2/3 and DG regions of the hippocampus are delineated by the red line, green line and blue line, respectively. Scale bar = 1 mm. **(B-C)** Illustrations of the unbiased counting method. The green line in the frame represents the inclusion line, and the red line in the frame represents the exclusion line. Iba1^+^ cells were counted when their somas were first focused within the disector height and their somas were completely inside the counting frame or only touching the inclusion line. **(B)** The Iba1^+^ cell soma was located in the counting zone (above the disector height) and was not in focus. Therefore, this cell was not counted. **(C)** The Iba1^+^ cell soma indicated by the arrow within the counting zone was counted. Scale bar = 10 μm.
