# Supplementary figures and images for: Beneficial effects of running exercise on hippocampal microglia and neuroinflammation in chronic unpredictable stress-induced depression model rats
Source: Transl Psychiatry. 2021 Sep 6;11:461. doi: 10.1038/s41398-021-01571-9 (PMC8421357; doi:10.1038/s41398-021-01571-9)

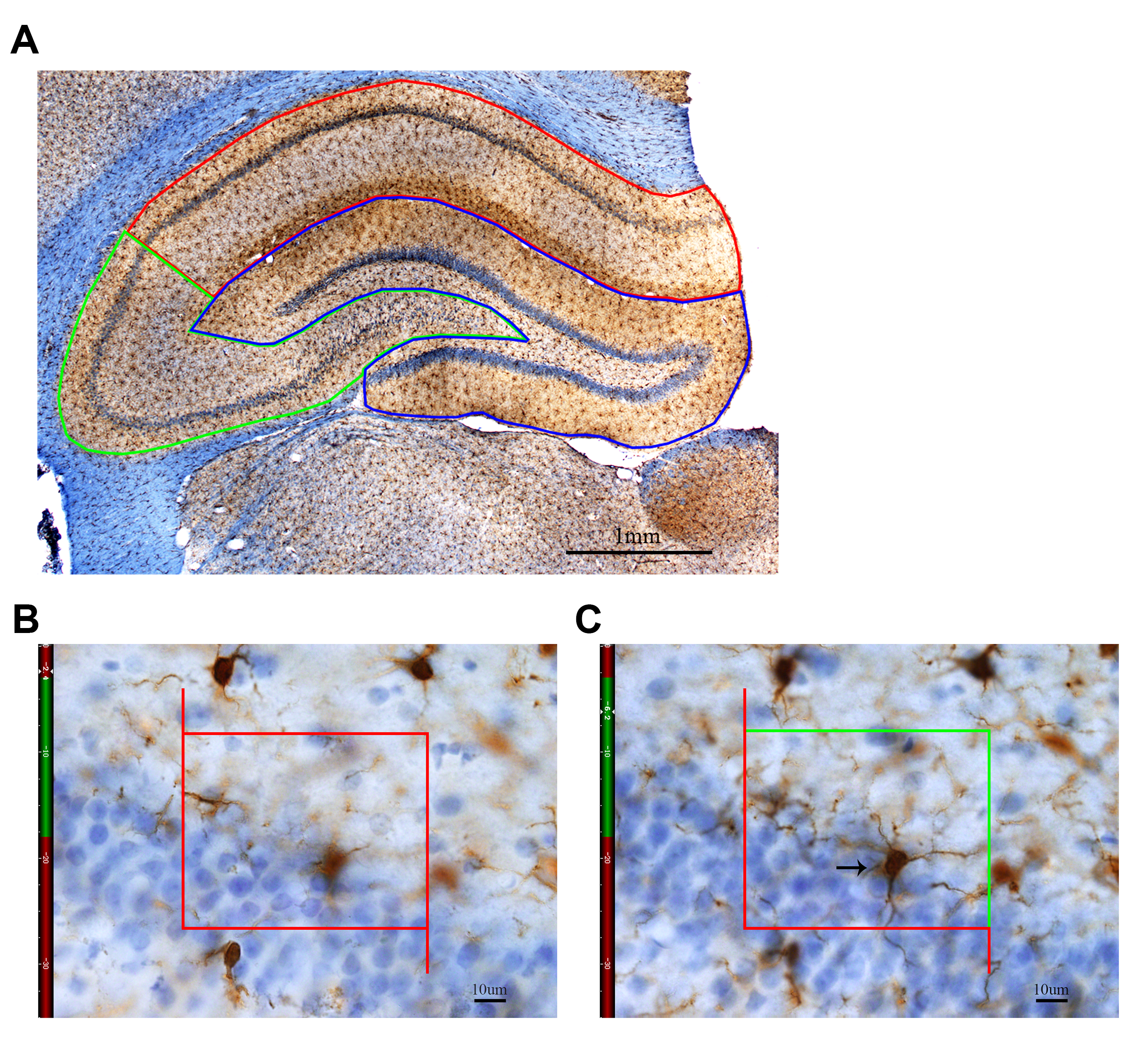

Supplement: Supplementary file 2 — Supplementary figure 1 [file 41398_2021_1571_MOESM2_ESM.tif]
